# Effect of Graphene Oxide on the Electrothermal and Pressure-Sensitive Properties of Carbon Fiber Cementitious Composites

**DOI:** 10.3390/ma17163928

**Published:** 2024-08-07

**Authors:** Jingjing He, Xuezhi Wang, Leiying Han, Siyue Wang, Ming Xin

**Affiliations:** 1 Power China Northwest Engineering Corporation Limited, Xi’an 710065, China; hejing_86@126.com; 2 School of Civil and Architectural Engineering, Liaoning University of Technology, Jinzhou 121000, China; hanleiying2022@163.com (L.H.); wsy1037992263@163.com (S.W.); xmmyemail@163.com (M.X.)

**Keywords:** graphene oxide, carbon fiber, cementitious composites, electrophoretic deposition, electrothermal heating, pressure-sensitive properties

## Abstract

The application of carbon fiber in cement matrix has some disadvantages, such as poor dispersion and poor interfacial adhesion. In order to improve the interaction between carbon fiber and cement matrix and improve the properties of cement-based composites, carbon fiber was modified by electrophoretic deposition of nano-graphene oxide (GO). In this paper, the effects of doping CF into the cement matrix before and after GO modification are studied comparatively in terms of electrical conductivity, electrothermal warming effect, and pressure-sensitive properties of the cement matrix. It was found that the GO-modified CF reduces both the electrical resistivity of cementitious composites and the required level of fiber incorporation compared to CF. The percolation threshold is 0.7 wt% for CF and 0.5 wt% for GO-CF. The GO-modified CF is more effective than CF as a conductive filler to enhance the electrothermal warming performance of the cement matrix. When the GO-CF doping rate is 0.5%, the specimen temperature increases most rapidly, and the temperature rise value reaches a maximum of up to 30.45 °C, which is twice that of the CF group. When the fiber content is 0.7%, the pressure sensitivity of the sample was the best. When the fiber content is 0.5%, GO-CF can improve the pressure sensitivity of cement mortar specimens, and increase the resistance change rate of the cement mortar specimens by 5.7%.

## 1. Introduction

With the continuous progress of time and technology, people have put forward more functional requirements for cementitious composites, which has accelerated the development of intelligent cementitious composites [1]. At present, conductive cementitious composites are still in the research stage in the field of construction, e.g., electromagnetic shielding [2,3], self-sensing [4,5], structural monitoring [6], electrothermal [7,8,9], etc. Carbon fiber (CF) is considered as a good reinforcing phase for electrothermal cementitious composites due to its excellent electrical and thermal conductivity, high strength, and durability [10,11].

Salam R. Armoosh et al. [12] investigated the electrical conductivity and electro-thermal properties of cementitious composites by using carbon fibers and carbon nanotubes as conductive packing materials, and the experimental results showed that the temperature of cementitious composites can reach up to 100 °C during the recording time when the input voltage is 60 V. Qin Zhaoqiao et al. [13] investigated the electro-thermal properties of nickel-plated carbon fiber (Ni-CF) cementitious composites, and the experimental results showed that Ni-CF improved the electro-thermal conversion of the cement matrix by up to 71.98%. Manuel Hambach et al. [14] found that Carbon Fiber Cementitious Composites (CFRC) can be used for temperature rise of interior walls as well as floors without the need for additional heating elements. Hongming Zhao et al. [15] proposed a de-icing method by embedding carbon fiber heating filaments in concrete slabs, which can be applied to melting snow and ice on pavements. According to the existing research, the electro-thermal properties of carbon fiber cementitious composites can effectively replace traditional methods of road salt de-icing and indoor air conditioning heating, and at the same time solve issues associated with these traditional methods, including heavy pollution, energy consumption and related problems.

However, due to drawbacks such as the difficulty of CF dispersion in the cement matrix and the poor bonding performance at the interface of CF and the cement matrix [16], the development of intelligent CFRC has been greatly limited. Therefore, seeking a better way to improve the dispersion of CF in aqueous solution and cement matrix, as well as how to modify CF to make its surface more active, is desirable for engineering applications.

The current methods for surface modification of CF include plasma treatment, electrophoretic deposition (EPD), and γ-ray treatment methods [17,18]. Graphene oxide (GO), a derivative of graphene, is a novel two-dimensional carbon nanomaterial [19] with a large number of hydroxyl, epoxy, carbonyl, and carboxyl groups on the surface and edges of its nanosheets. These oxygen-containing functional groups are highly hydrophilic, which can lead to better dispersion of GO in water [20,21]. GO has excellent mechanical properties, and the tensile strength of multilayer GO can be as high as 130 MPa [22]. Meanwhile, there is good bonding between GO and cement matrix, which further improves the mechanical properties of cementitious composites. A large number of scholars have doped GO into the cement matrix to study the effect of GO on the properties of cementitious composites [23,24,25]. Lv et al. showed that GO can have good bonding with cementitious composites and makes the hydration reaction products of cement turn into flower-like crystals to improve the pore structure of the composite, thus enhancing its mechanical properties of [26]. On the other hand, using a molecular dynamics simulation method, Wang et al. found that the abundant functional groups on the surface of GO enhanced the interfacial binding properties between GO and CSH [27]. In addition, GO can be prepared from graphite by the modified Hummers method at a low cost, which provides a good basis for its widespread use. Therefore, GO is considered to be a good material for the modification of CF surfaces.

To date, there is no study using graphene oxide carbon fiber hybrid fibers to evaluate the electro-thermal properties of cementitious composites. Therefore, in this study, graphene oxide carbon fiber hybrid fibers (GO-CF) were prepared by modifying the CF surface with GO using the EPD method to introduce reactive groups on the CF surface. The effects of GO-CF and CF as conductive fillers on the electrical conductivity and electrothermal properties of cementitious composites were comparatively investigated, and the mechanism of their action was additionally analyzed from the microstructural perspective. It is of great theoretical value to improve the electrical conductivity as well as the electrothermal properties of cementitious matrix using GO-CF hybrid fibers, and the results are expected to be applied in the field of smart cements.

## 2. Materials and Methods

### 2.1. Raw Material

P·O 42.5 grade ordinary silicate cement was used. The main components are shown in Table 1 and physical properties are shown in Table 2. The sand adopted was graded river sand with a maximum particle size of 2 mm; the polycarboxylic acid water reducing agent (PC) was produced by Sinopharm Chemical Reagent Co., Ltd. (Shanghai, China), with a solid content of 45 wt%; CF was provided by Toray Carbon Fibers (Guangdong) Co., Ltd. (Guangzhou, China); and GO was provided by Xiamen Knano Graphene Technology Co., Ltd. (Xiamen, China), with a thickness of 5 nm and a lamella diameter of 5–10 µm.

### 2.2. Preparation of GO Electrolyte

**Figure 1:**
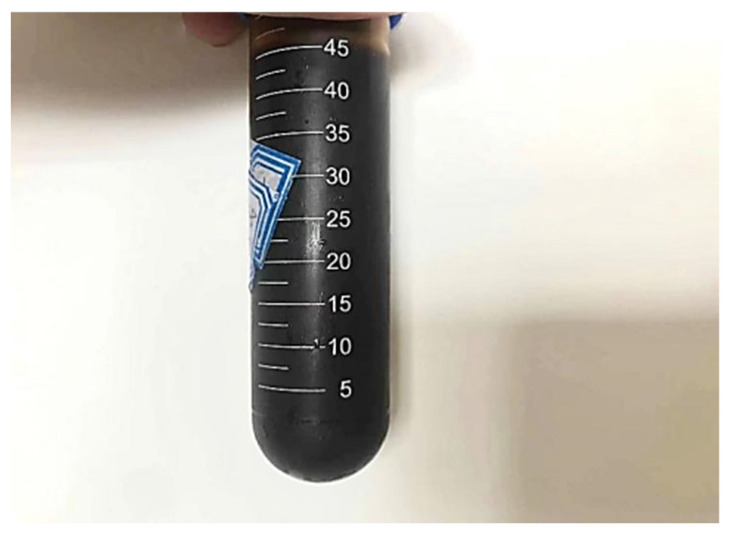
GO solution.

A quantitative amount of GO and deionized water were placed in a tank sonicator and dispersed by ultrasonication for 1 h to prepare GO electrolyte of 1.5 mg/mL concentration, which was stored for later use. The configured GO electrolyte is shown in Figure 1.

### 2.3. Preparation of GO-CF by Electrophoretic Deposition

**Figure 2:**
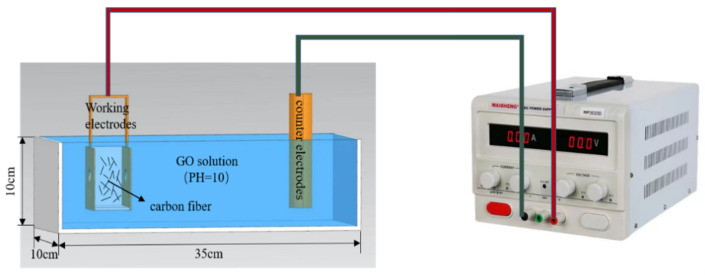
Diagram of EPD.

The schematic diagram of electrophoretic deposition is shown in Figure 2. The working electrode used a copper box, which was filled with CF. Utility tools were used to drill two holes on both sides of the copper box, and a filter was placed in the holes to ensure that CF cannot leak out of the copper box when the CF was in full contact with the GO electrolyte. The counter electrode was a copper plate, with the distance between the two being fixed at 5 cm. An MP3030D type DC power (MAI SHENG Corporation, Suzhou, China) supply was used. The surface of commercial carbon fibers was coated with a layer of commercial sizing agent [28,29], which affects the wrapping of CF by GO. Therefore, the surface of CF should be treated before modification. NaOH solution was added to the GO solution; the test was carried out under constant temperature, the voltage of the DC regulated power supply was adjusted to 15 v, the pH value of the GO electrolyte was adjusted to 10.0, the concentration was 1.5 mg/mL, and the modification time was 40 min. After deposition, the modified CF was rinsed with deionized water and then dried naturally at room temperature to obtain the GO-modified CF (GO-CF).

### 2.4. Design of Mix Ratio and Specimen Production

According to the provisions of “Test method of cement mortar strength (ISO method) (GB/T 17671-2021)” [30], the specimen size was 40 × 40 × 160 mm. Three specimens were designed for each group; the results were averaged, and a WDW-300 electronic universal testing machine (Changchun Kexin Experimental Instrument Co., Ltd., Changchun, China) was used. The microstructure of cementitious composites was analyzed using an electron scanning microscope (Zeiss Sigma500, Oberkochen, Germany). Copper mesh electrodes were used. The fiber dosages were 0.1 wt%, 0.3 wt%, 0.5 wt%, 0.7 wt%, 1 wt%, and 1.5 wt% of the cement mass, and methyl cellulose accounted for 20% of the fiber mass. The specific mix ratio is shown in Table 3. The specimens were prepared by dispersing CF and GO-CF in weighed water containing methyl cellulose, with an appropriate amount of PC added and mixed well. The weighed PC was poured into a cement mortar mixer, stirred together with cement and sand, stirred evenly according to the cement mortar mixing standard, and poured into a test mold of 40 × 40 × 160 mm for insertion and ramming; then it was placed on a vibration table for compaction. Four pieces of copper mesh electrode were inserted in parallel the long side of the specimen, and the specimen was demolded after 24 h. The experimental specimens were dried at room temperature for 48 h (the average moisture content of the tested specimens was 3.07%) and then tested for functionality to ensure the reliability and authenticity of the data.

### 2.5. Experimental Method

#### 2.5.1. Conductivity Test

**Figure 3:**
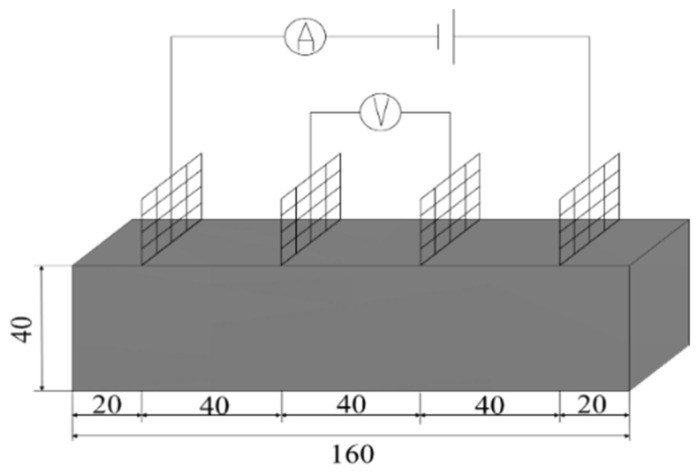
Schematic diagram of four-electrode resistivity test.

**Figure 4:**
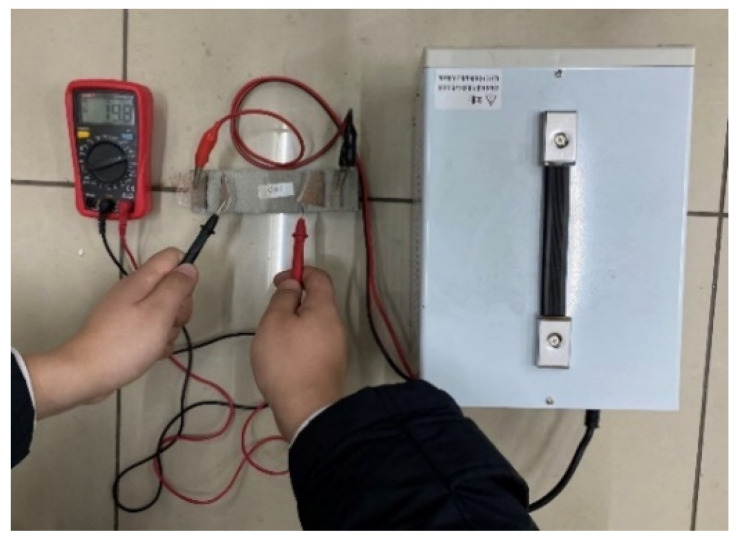
Resistivity test.

Resistivity is an important parameter to characterize the electrical conductivity of materials. In order to reduce the influence of the polarization effect on the experimental results, the four-electrode method was used to test the resistivity. An MP3030D DC regulated power supply was used to supply power, while a digital multimeter was used to test the voltage at both ends of the specimen. The two copper grids outside the specimen are current poles and the two copper grids inside are voltage poles. The test schematic is shown in Figure 3 and the test procedure is shown in Figure 4.

The resistivity of the specimen is calculated according to the following formula:

*R* = *U*/*I* (1)

*ρ* = *S*/*L* × *R* (2)

where *R* is the resistance of the test piece to be tested (Ω); *U* and *I* are the voltage (V) and current (A) at the two ends of the test piece to be tested, respectively; *ρ* is the specimen resistivity (Ω·m); *S* and *L* are the area of the internal electrode of the mortar (m^2^) and the distance between the two electrodes of the inner side (m), respectively.

#### 2.5.2. Electrothermal Temperature Rise Performance Tests

**Figure 5:**
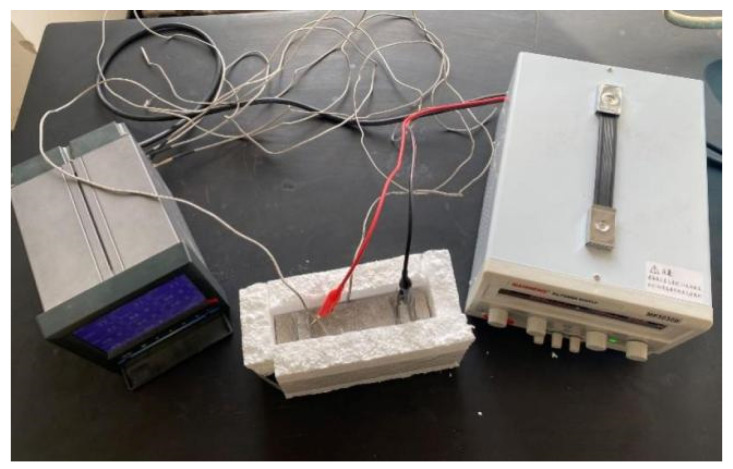
Diagram of electric heating test device.

A DC regulated power supply was used to apply a voltage of 30 V to both ends of the specimens, and the surface temperature of the specimens was collected by a paperless recorder at 90 s intervals, recorded for 15 min under constant power conditions. The specimens were placed in a freezer for refrigeration before the test, and the temperature of the specimens was lowered to 0 °C. In order to reduce heat loss, an experimental process was used to insulate the benzene board wrapped around the specimen. The experimental setup diagram is shown in Figure 5.

#### 2.5.3. Pressure-Sensitive Performance Test

**Figure 6:**
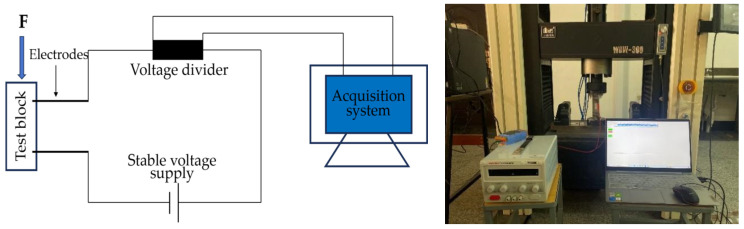
Pressure sensitivity test loading device.

The schematic diagram of the pressure sensitivity test is shown in Figure 6. The test was carried out using a WDW-300 electronic universal testing machine. The upper and lower surfaces of the specimen were padded with insulating plates, and the voltage dividing resistor in the device was a 100 Ω constant value resistor. An MP3030D DC regulated power supply was used to supply power, and the power supply voltage was set to 20 V. According to the principle of series circuit voltage division, the resistance value *R* of the specimen to be tested can be calculated by Formula (3). During the test process, the stress change value and voltage change value of the voltage divider resistor were collected, and the resistance change rate was calculated according to Equation (4) at the end of the test.

(3) R=(U/Ux−1)Rx

where *U* and *Ux* are the voltage of the power supply and the voltage of the voltage divider collected by the system, respectively; *Rx* is the resistance value of the voltage divider.

(4) $$\Delta \rho =(R-R_{0})/R_{0}$$

where *R* and *R*_0_ are the resistance value of the specimen at any moment during the loading process and the stable resistance value of the specimen before loading, respectively.

## 3. Results and Discussion

### 3.1. Characterization of CF before and after Modification

**Figure 7:**
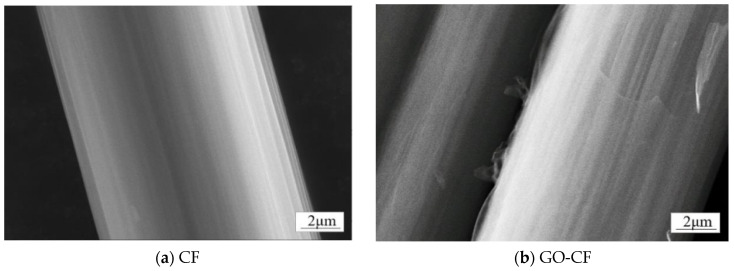
SEM images of the surface morphology of CF and GO-CF hybrid fibers.

In order to determine the conversion of CF to GO-CF, SEM was used to analyze the morphological changes of both. As shown in Figure 7a, the surface micromorphology of deslurried CF is flat and smooth. Figure 7b shows the CF modified with GO; compared with the unmodified CF, the GO covers the CF surface, making the GO-CF surface rougher.

**Figure 8:**
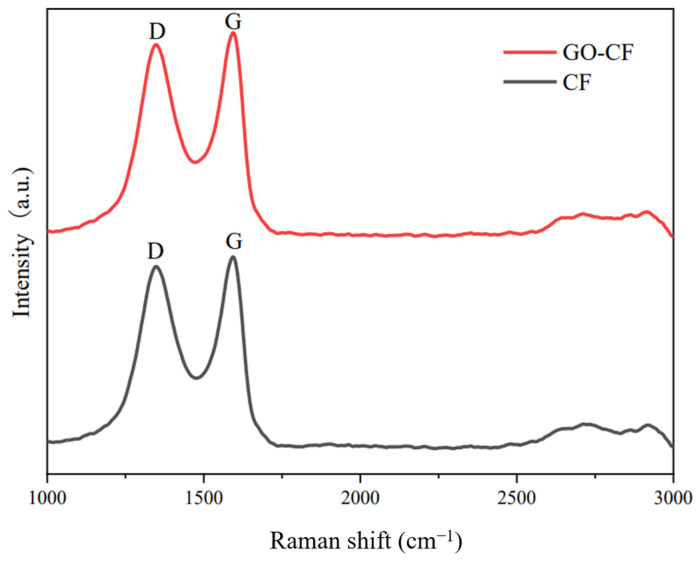
Raman spectra of CF before and after GO modification.

The Raman spectra of CF and GO-CF are shown in Figure 8. From the figure, it can be seen that there are two characteristic signal bands in the Raman spectra of both CF and GO-CF. The D band is the result of the induction of disordered structure, and the G band is the result of the induction of ordered structure. The intensity ratios of the D and G peaks, ID/IG, are generally used to illustrate the apparent conditions of carbonaceous materials [31]. The ID/IG values of GO-CF and CF were 0.87 and 0.82, respectively, indicating an increase in the disordered structure of the CF surface after GO modification, suggesting that the oxygen-containing functional groups carried by GO made the structure of the CF surface more disordered, thereby proving that GO had been successfully introduced into the CF surface.

**Figure 9:**
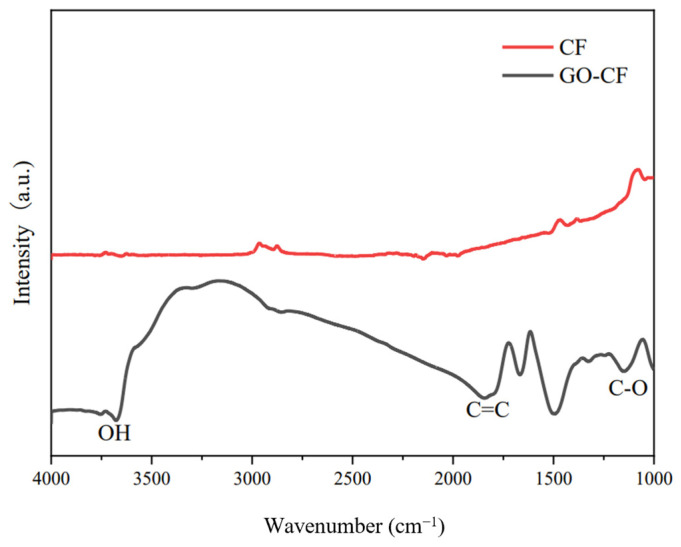
FTIR diagram of CF before and after GO modification.

**Figure 10:**
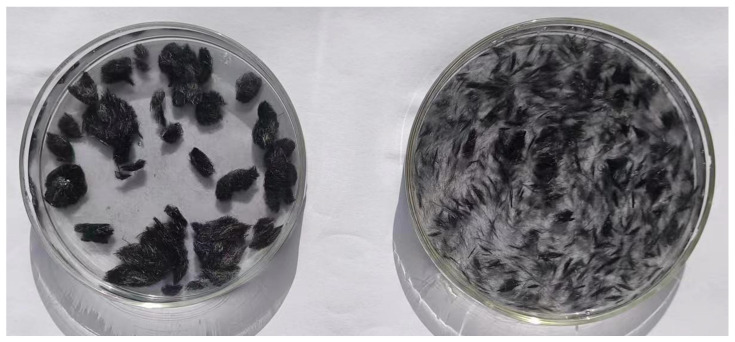
Dispersibility of CF in water before and after modification ((**left**) is unmodified, (**right**) is modified).

Figure 9 shows the infrared spectra of CF before and after GO modification. From the picture we can see that there is almost no peak in the infrared spectrum of CF, which indicates that CF is chemically stable and has almost no functional groups on the surface. In contrast, many peaks appear in the infrared spectrum of the GO-modified CF, which indicates that a large number of oxygen-containing functional groups appear on the surface of the GO-modified CF, such as the -OH stretching peaks, carbon-carbon double bond stretching peaks, etc. The appearance of these peaks indicates that GO has successfully modified the CF, and introduced oxygen-containing functional groups into the CF surface. This helps to improve its partitioning activity in the cement matrix, which helps to improve its dispersion in the cement matrix and water, as shown in Figure 10.

### 3.2. Electrical Conductivity of GO-CF Cementitious Composites

There are three main mechanisms of CF conductivity in cementitious composites: ionic, electronic, and hole conductivity [32].

**Figure 11:**
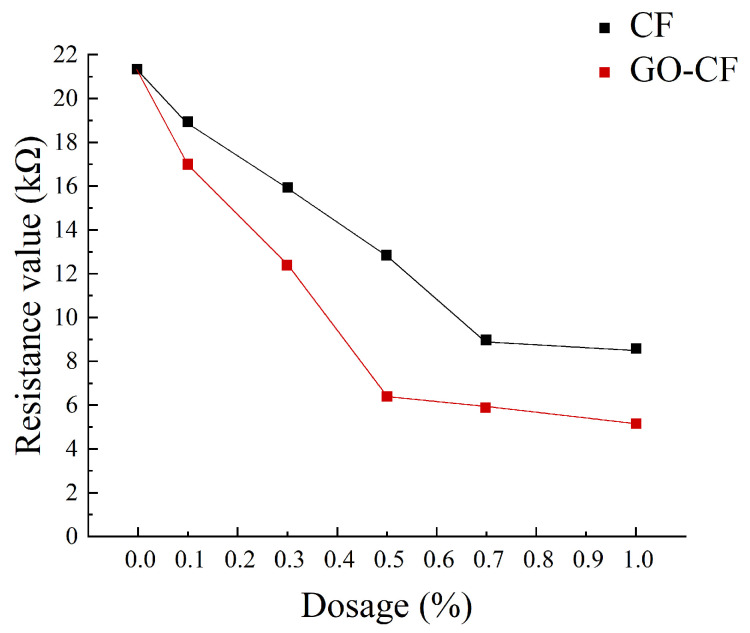
Resistance curves with different fiber content.

**Figure 12:**
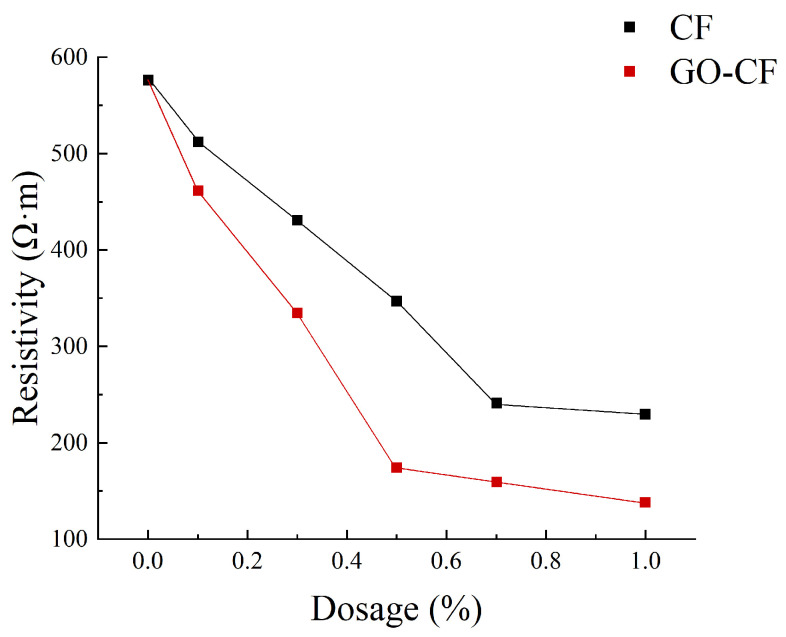
Resistivity curves with different fiber content.

Figure 11 shows a graph of the measured resistance variation with fiber doping, and Figure 12 shows the resistivity variation of CF and GO-CF cementitious composites. As can be seen in Figure 11 and Figure 12, the resistivity of cementitious composites shows an overall decreasing trend with increasing CF doping. When CF doping was in the range of 0–0.3 wt%, the resistivity decreased significantly, by 25.35% compared to pure cement. When the CF doping was in the range of 0.3–0.7 wt%, the resistivity decreased sharply, and the percolation threshold was reached when the CF doping was 0.7 wt%. When the CF doping exceeded 0.7 wt%, the resistivity did not change significantly with the increase of fiber doping and basically became stable. The overall trend of resistivity change of GO-CF cementitious composites is the same as that of CF cementitious composites: with the increase of GO-CF doping, the resistivity decreases gradually and finally tends to be stable. However, it is obvious from the figure that the resistivity of GO-CF cementitious composites decreased more rapidly and to a greater extent. At the same time, the percolation threshold of GO-CF doping was reduced to 0.5 wt%, and when the GO-CF doping was 0.5 wt%, the resistivity saw a maximum decrease to 48.39%, which was 69.95% compared with pure cement.

**Figure 13:**
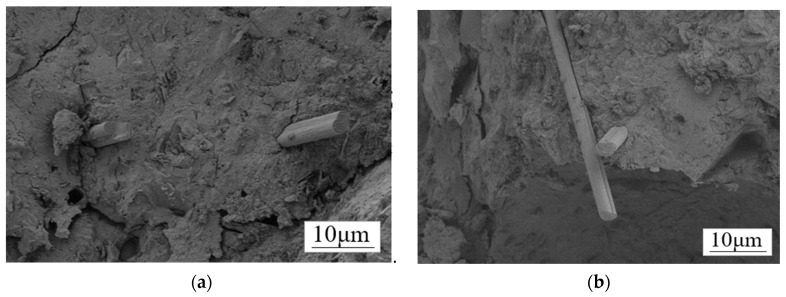
SEM morphology of CF/GO-CF cement matrix composites: (**a**) the CF is separated by large concrete blocks that cannot be interlocked; (**b**) GO-CF fibers overlap each other in the cement matrix.

This phenomenon arises because, when the fiber doping is low (0~0.3 wt%), the fibers are widely spaced in the cement matrix, with almost no overlap with each other, and the carriers cannot flow from the end with high electric potential to the low end. At this time, the cement matrix is mainly ionic-conductive through the ions in the pore solution (e.g., OH^−^, K^+^, Na^+^, and Ca^2+^), so the effects of GO-CF and CF on the electrical resistivity of cementitious composites are almost the same. When the fiber doping is gradually increased, the spacing between fibers is reduced, lapping between fibers occurs, forming a conductive network, and electrons will flow from the end with high electric potential to the low end, at which time ionic conductivity and electronic conductivity occur at the same time, and the resistivity decreases rapidly. However, the resistivity of GO-CF cementitious composites decreased more rapidly and more than that of CF cementitious composites. This is because the surface of the GO-modified CF has a large number of oxygen-containing functional groups, which makes the surface of CF more active and the interaction between CF and cement matrix is strengthened. At the same time, GO effectively improved the dispersion of CF in the cement matrix, increased the mutual overlap between fibers, and enhanced its electronic conductivity. From the SEM image in Figure 13, it can be clearly observed that CFs are poorly dispersed in the cement matrix and easily appear in bundles. They were separated by the bulk cement and could not overlap one another. On the other hand, GO-CF is uniformly dispersed in the cement matrix and the fibers overlaps one another to form a conductive network. When the fiber doping is too much, the fiber doping has reached the percolation threshold, the fibers overlap each other to form a complete conductive network, and the resistivity almost does not change with further fiber doping. π electrons cross the potential barrier between two adjacent fibers, and leap from one fiber to the adjacent fiber to form a tunneling effect [33], and at this time, hole conductivity dominates. The percolation threshold is 0.7 wt% for CF and 0.5 wt% for GO-CF, which is due to the fact that the dispersion of GO-CF in the cementitious matrix is much better than that of CF, and a complete conductive pathway has been formed when the dosage is 0.5 wt%. Although the resistivity change has stabilized at this stage, it is clear from Figure 12 that the resistivity of GO-CF cementitious composites is lower than that of CF cementitious composites. This is due to the fact that the CF agglomerates and is in a bundled state, and there is a cement barrier between each bundle of CF, which makes the distance between the CFs greater, resulting in prevention of the tunneling effect.

From the experimental results and the above analysis, it can be seen that CF modified by GO not only reduces the electrical resistivity of cementitious composites even further, but also reduces the required level of fiber doping compared to CF.

### 3.3. Electrothermal Warming Properties of GO-CF Cementitious Composites

**Figure 14:**
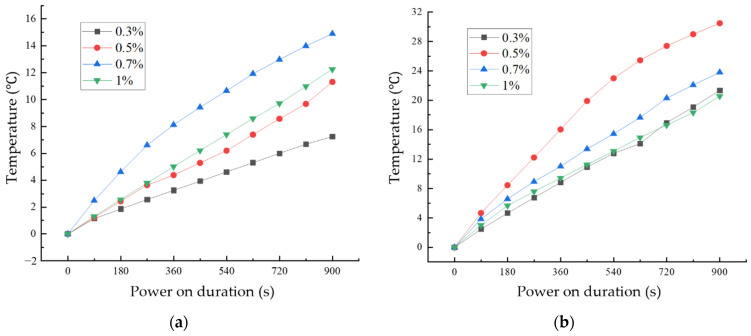
Temperature rise curves of cement-based composites with different fiber content: (**a**) CF cementitious composites temperature rise; (**b**) temperature rise pattern of GO-CF cementitious composites.

Figure 14 shows the variation of temperature with time for CF cementitious composites and GO-CF cementitious composites under energized conditions with different fiber dosages. From Figure 14a, it can be seen that the surface temperatures of CF cementitious specimens all increased with the increase of energization time. Comparing the four fiber dosages, when the CF dosage was 0.7%, the surface temperature of the specimen increased most rapidly and the temperature rise value was the largest, and the surface temperature of the specimen reached 14.89 °C when energized for 900 s.

From Figure 14b, we can see the change rule of specimen temperature with energization time under different GO-CF doping. With the extension of energization time, the surface temperature of specimens with different GO-CF doping increase. When energized for 720 s, the real-time temperatures of the specimens with 0.3% and 1% fiber doping were the same; when the GO-CF doping was 0.5%, the temperature of the specimen increased most rapidly and the temperature rise was the largest, and when energized for 900 s, the surface temperature of the specimen could reach 30.45 °C, which was twice as much as the maximum temperature rise of CF cementitious composites.

According to Joule’s law Q = (U^2^/R)t, the heat generated by each specimen after energization varies due to differences in resistance. Electrothermal heating performance means that the heat generated by cementitious composites under energized conditions increases gradually with the extension of the energized time, that is, the process of converting electrical energy into heat energy, which makes the temperature of the specimen increase.

Analysis of CF cementitious composites electrothermal warming mechanism: (1) The free Ca^2+^ and OH^−^ ions in the liquid phase of the cement matrix move directionally under the action of the external electric field, and collision occurs in the process of movement to release energy, thus converting electrical energy into heat energy; (2) the hydrated gel in the cement matrix as well as unhydrated cement particles in the valence of the electrons can be electrically conductive. In the conductive process, the electrons collide with each other to release energy, which converts electrical energy into thermal energy. (3) CF has good electrical conductivity and thermal conductivity, and heat energy is generated through the formation of pathways between CFs.

Analysis of the mechanism of GO-CF in cement matrix on the role of electrothermal warming: GO improves the dispersion of CF in cement matrix to a large extent, and increases the mutual overlap between CFs, thus forming conductive pathways, and these macroscopic pathways increase the current flow and heating efficiency; on the other hand, GO itself carries OH-plasma, which under the condition of energization undergoes directional movement, and collisions also generate a certain amount of heat. In addition, the poor bonding between CF and the cement matrix will produce more pores and increase the pore size of the structure, while after GO modification of CF, many hydration products will be attached to the surface, which increases the degree of bonding between CF and the cement matrix, thus reducing the existence of pores to a large extent and improving the heating efficiency of cement matrix composites. With increased fiber doping, the rate of warming on the surface of the specimen as well as the maximum temperature rise value decreased, which is due to the fact that when the doping of GO-CF exceeds 0.5%, the dispersion of the fibers in the cement matrix decreases, and the conductive effect becomes poorer, therefore the electrothermal warming effect decreases.

The electrothermal warming effect is due to the carriers in the cement matrix under energized conditions producing directional movement under the action of the external electric field, and the carriers colliding with each other to release energy, thus converting electrical energy into thermal energy [34]. When the doping of the conductive phase is small, as described in Section 2.1, the carriers cannot flow from the high electric potential end to the low end, and at this time the cement matrix exhibits mainly ionic conductivity; when the doping of the conductive phase reaches the percolation threshold, resulting in the formation of a connected conductive network, ionic conductivity and electronic conductivity occur at the same time, and the thermal conductivity is mainly affected by the directional movement of the free electrons. In the previous subsection, according to the change of resistivity, the GO-CF cement matrix percolation threshold is 0.5%. At 0.3% GO-CF doping, the cement matrix has not yet reached the percolation threshold; at this time, ionic conductivity and hole conductivity constitute the main mechanisms of electrical conductivity, and the energy released by the electronic energy state change is much smaller than the heat generated by the collisions between electrons. Therefore, although the specimen’s electrical resistance value is large, the rate of temperature rise and the maximum temperature rise value is low. When the doping amount reaches the percolation threshold of 0.5%, ionic conductivity and electronic conductivity become the main modes of conductivity, and the rate of temperature rise and the maximum value of temperature rise reach their maximum values.

It can be seen from Section 2.1 that the percolation threshold of CF in cement matrix is 0.7%, which is higher than that of GO-CF in cement matrix, and the maximum temperature rise value as well as the rate of temperature rise of CF cement matrix are lower than that of GO-CF cement matrix. Therefore, the GO-modified CF is more effective as a conductive filler to enhance the electrothermal warming performance of the cement matrix.

### 3.4. Pressure-Sensitive Properties of GO-CF Cementitious Composites

**Figure 15:**
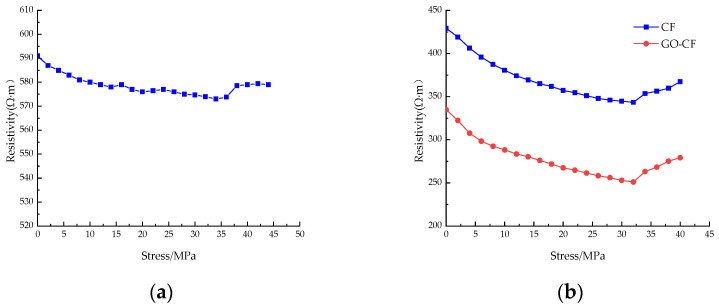

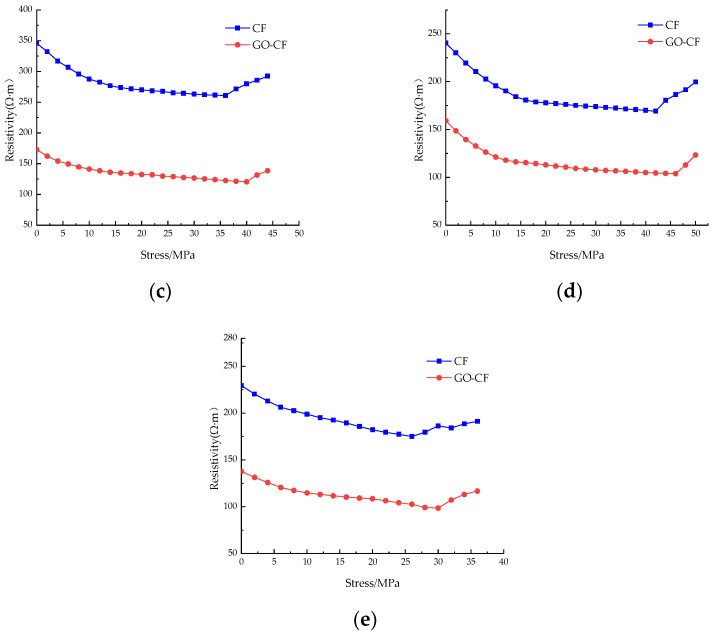
The resistivity curve of CF/GO-CF cement mortar specimens varies with stress. (**a**) PC; (**b**) 0.3%CF/GO-CF; (**c**) 0.5%CF/GO-CF; (**d**) 0.7%CF/GO-CF; (**e**) 1%CF/GO-CF.

Figure 15 shows the resistivity curves of CF cement mortar specimens and GO-CF cement mortar specimens under stress with different fiber content. You can see from the picture that under monotonic loading, the resistivity of ordinary cement mortar specimens is almost unchanged, and only slight fluctuation occurs. The resistivity change is poor in regularity, and there is no rule to follow; when doped with CF/GO-CF, the resistance of cement mortar specimens with different fiber contents decreases first and then increases with the increase of applied stress, and the resistivity decreases rapidly in the early stage of stress application.

When a certain amount of fiber is included, the resistivity of GO-CF cement mortar specimens decreases more than that of CF cement mortar specimens. When the fiber dosage was 0.3%, fluctuation was obvious during the curve’s decline, and there was a significant decrease compared with the PC group. The resistivity of the CF cement mortar specimen decreased by 20%, and that of the GO-CF mortar specimen decreased by 25%, which is 5% higher than the CF mortar specimen. When the fiber doping was 0.5%, the decrease in resistivity began to increase with increasing pressure. The resistivity of the CF cement mortar specimen decreased by 24.5%, and the resistivity of the GO-CF mortar specimen decreased by 30.2%, which is 5.7% higher than that of the CF mortar specimen. When the fiber doping was 0.7%, the resistivity of CF cement mortar specimens decreased by 29.6%, and the resistivity of GO-CF mortar specimens decreased by 34.7%, which is 5.1% higher than that of the CF mortar specimens. Here, the decrease in resistivity of CF cement matrix has begun to decrease compared with specimens dosed at 0.5%, whereas the decrease in resistivity of the GO-CF cement matrix was still increasing. When the fiber doping was 1%, the resistivity of the CF cement mortar specimen decreased by 23.7% and that of the GO-CF mortar specimen decreased by 28.4%. Compared with the sample of CF cement mortar, this is an increase of 4.7%. Here, the sensitivity of the specimen began to decrease and its pressure sensitivity began to deteriorate.

When the applied external stress was low, the fine cracks and pores inside the specimen gradually closed and the internal fibers of the specimen were lapped together under the action of stress extrusion to form a conductive network, so the electrical resistivity decreased rapidly under the action of stress; when the applied load reached a certain value and the original cracks and pores were fully closed, new cracks and pores were generated inside the specimen, which began to fail. Meanwhile, the fibers inside the specimen were further squeezed together, and part of the conductive network failed. Therefore, the resistivity decreased slowly with the increase of stress. When the applied stress reached the limit, the specimen failed completely, the conductive network was damaged, and the resistivity increased sharply.

In summary, with certain fiber content, the pressure-sensitive properties of the cement-based composite with GO-modified CF are better than those of the unmodified CF cement-based composite. The best pressure-sensitive properties of the specimens are obtained when the fiber doping is 0.7%; the best improvement of pressure-sensitive properties of the mortar specimens is achieved by GO-CF when the fiber doping is 0.5%, and the change rate of electrical resistance is improved by 5.7% compared with that of CF cement mortar specimens.

It can be seen from this experiment that GO-CF cementitious composites have good pressure-sensitive properties, and the dosage of CF can be reduced after GO modification. This not only reduces the cost, but also significantly improves the pressure-sensitive effect, so the composites are expected to be applied to an intelligent cement base. A sensor made of GO-CF cementitious composites can be embedded in a structure to form a detection system. In the event of a pressure overload, an alarm will be given, capable of detecting the structural failure and thus ensuring safety.

### 3.5. Microstructure Analysis

**Figure 16:**
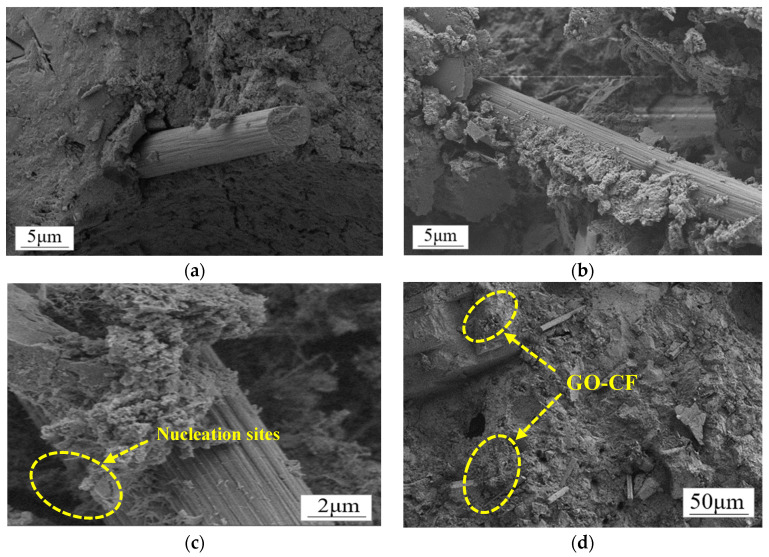
SEM morphology of CF/GO-CF cement matrix composites. (**a**) Interfacial microstructure of CF cement matrix. (**b**) Microstructure of GO-CF cement matrix interface. (**c**) Microstructure of GO-CF cement matrix interface. (**d**) Dispersion of GO-CF in the cement matrix.

Figure 16 shows the SEM morphology of CF and GO-CF in cement matrix. The functional changes of CF and GO-CF were analyzed by SEM. Compared with ordinary cement mortar specimens, CF cement mortar specimens have improved electrical conductivity, electrical and thermal heating performance, and pressure sensitivity, mainly because CF itself has very good electrical and thermal conductivity, which can effectively improve the function of the cement matrix. The sensitivity to electrothermal warming changes of the cement matrix and its pressure-sensitive properties depend mainly on the dispersion of CF in the cement matrix, where well-dispersed CF has a more connected conductive network, and also on the interaction between the CF and the cement matrix.

Figure 16a clearly shows that there is a large void at the interface of CF and the cement matrix, the surface of the CF is smooth, there is no wrapped hydration product on the surface, and the bonding performance at the interface of the CF and cement matrix is poor. Therefore, the interface between CF and cement matrix cannot produce a good interaction, which strongly affects the functionality of the cement matrix. Figure 16b shows the SEM morphology of the interface of GO-CF and cement matrix. From the figure, it can be clearly seen that there are a large number of cement hydration crystals encapsulated on the surface of the CF, which greatly improves the bonding performance at the interface of CF and the cement matrix, and enhances the interaction between CF and the cement matrix. The voids at the interface of CF and the cement matrix are filled up, which leads to a denser microstructure, which improves the functionality of the cement matrix at the macro level.

Figure 16c shows more clearly that GO provides nucleation sites for cement hydration crystals on the CF surface. The surface of CF modified with GO contains a large number of oxygen-containing functional groups, which provide nucleation sites for the cement hydration products and play the role of template, making the surface properties of CF more active. At the same time, the GO on the CF surface also has a bridging effect, which makes the CF and cement hydration crystals closely connected, effectively improving the physical friction and chemical bonding effect on the CF surface, and makes the interaction at the interface of CF and the cement matrix more adequate. On the other hand, the large number of oxygen-containing functional groups on the CF surface further improves the dispersion of CF in the cement matrix, which more easily produces overlapping and the formation of a connected conductive network, thus improving the functionality of the cement matrix.

**Figure 17:**
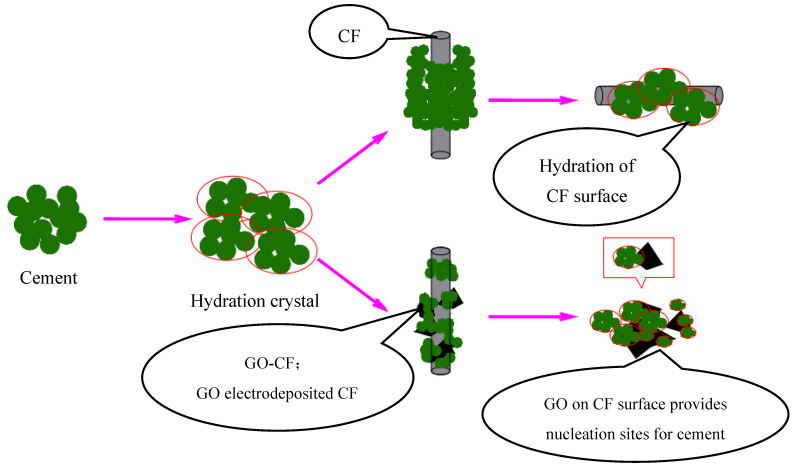
Schematic illustration of hybrid effect of GO-CF on cement material.

In Figure 17, the interaction with cement hydration crystals before and after CF modification is shown schematically. It can be clearly seen that due to the smooth surface of CF, there is a large void between the cement hydration crystals and CF surface, and the bonding performance between them is poor. On the other hand, on the surface of GO-CF hybrid fibers, GO provides nucleation sites for cement hydration crystals and has a regulatory effect on cement hydration products. The hydration crystals are aggregated and embedded in the GO surface, which improves the bonding performance at the interface of CF and the cement matrix, and also improves the dispersion of CF in the cement matrix, which gives the GO-CF cementitious composites good functionality.

On the other hand, the GO-CF hybrid fibers have strong electrostatic repulsion and spatial stability, and thus the dispersion of GO-CF in the cement matrix is superior to that of CF in the cement matrix. As can be seen from Figure 16d, GO-CF can be well dispersed in the cement matrix, and this good dispersion is a prerequisite for inducing CF to form a conductive network in the cement matrix. Under the action of external force, the well-dispersed CF in the cement matrix is extruded by the external force, causing fibers to overlap each other to form a conductive network. Therefore, GO-CF cementitious composites are significantly better than CF cementitious composites in terms of electrical and thermal warming properties and pressure-sensitive properties.

## 4. Conclusions

In this paper, the effects of doping CF into cement matrix before and after GO modification are studied comparatively in terms of electrical conductivity, electrothermal warming effect, and pressure-sensitive properties of the cement matrix, and the following conclusions are given:

(1) The GO-modified CF not only further reduces the electrical resistivity of cementitious composites, but also reduces the required level of fiber incorporation compared to CF. Percolation thresholds are 0.7 wt% for CF and 0.5 wt% for GO-CF.
(2) The GO-modified CF is more effective than CF as a conductive filler to enhance the electrothermal warming performance of the cement matrix. When the GO-CF doping is 0.5%, the temperature of the specimen increases most rapidly and the temperature rise value is the largest, and the surface temperature of the specimen can reach 30.45 °C after 900 s of energization, which is twice as much as the maximum temperature rise of CF cementitious composites.
(3) Under monotonic loading, the resistivity of both CF and GO-CF cement mortar decreased gradually, and the rate of decrease was faster in the early stage, later becoming slower. Compared with the CF cement mortar specimen, the resistivity of the GO-CF cement mortar specimen decreased more. This indicates that the GO-CF cement mortar specimens have better pressure-sensitive properties. The best pressure-sensitive performance of the CF specimens was obtained when the fiber dosage was 0.7%. The best improvement in pressure-sensitive performance of mortar specimens was achieved by GO-CF when the fiber doping was 0.5%, which increased the resistivity change by 5.7% compared to CF cement mortar specimens.

## Figures and Tables

**Table 1:** The main components of P·O 42.5 grade ordinary Portland cement.

Main Content	CaO	SiO_2_	Al_2_O_3_	Fe_2_O_3_	SO_3_	MgO
Ratio (wt%)	66.30	19.60	6.50	3.50	2.50	0.70

**Table 2:** Physical properties of P O 42.5 cement.

Ignition Loss (%)	Initial Setting/Time (min)	Final Setting/Time (h)	Specific Surface Area/(m^2^⋅kg^−1^)	Flexural Strength/MPa		Compressive Strength/MPa	
				3 d	28 d	3 d	28 d
≤5	180	6	351	6.0	8.4	30.4	53.6

**Table 3:** Mix ratio of GO-CF cement mortar.

Cement/kg	Water/kg	Sand/kg	PC/kg
560	210	1120	2.8

## Data Availability

The original contributions presented in the study are included in the article, further inquiries can be directed to the corresponding authors.
